# Generalized Anxiety disorder but not depression is associated with insomnia: a population based study

**DOI:** 10.5935/1984-0063.20180031

**Published:** 2018

**Authors:** Imran Wasfi Khan, Ruchi Juyal, Deep Shikha, Ravi Gupta

**Affiliations:** 1 Himalayan Institute of Medical Sciences, Community Medicine - Dehradun - Uttarakhand - India.; 2 Himalayan Institute of Medical Sciences, Psychiatry - Dehradun - Uttarakhand - India.

**Keywords:** Insomnia, Epidemiology, Prevalence, Mental Disorders

## Abstract

**Background:**

Insomnia is a common problem, however, its prevalence has never been examined in Indian population. Moreover, a number of psychiatric disorders have been found to be associated with insomnia in clinical population, but this association has scarcely been examined in general population.

**Methods:**

This epidemiological study was done in an urban and a rural population. Subjects were selected using Kish method. After obtaining informed consent, psychiatric disorders were diagnosed using Hindi version of Mini International Neuropsychiatric Interview. Hindi version of Insomnia Severity Index was used to diagnose insomnia.

**Results:**

1700 subjects were included in this study. In this study, prevalence of insomnia was 10.3%. Its prevalence increased with increasing years of education (*p*=0.009). Insomnia was more frequent in subjects living in joint families (*p*<0.001), having higher education (p=0.009), those who were separated (*p*<0.001), among subjects belonging to middle socio-economic status (*p*<0.001) and in urban population compared to semi-urban and rural population (*p*<0.001). Insomnia was also more frequent among subjects with major depressive disorder, generalized anxiety disorder, alcohol dependence, cannabis dependence and tobacco use. However, binary logistic regression analysis showed that only higher education, unemployment, generalized anxiety disorder and tobacco use were associated with insomnia.

**Conclusion:**

Insomnia in general population is associated with higher education, unemployment, generalized anxiety disorders and tobacco use.

## INTRODUCTION

Insomnia is a common complaint in patients attending Psychiatry clinics with nearly four fifth reporting disturbed sleep^1^severity of the same was determined. RESULTS 83.4% of the population had some type of sleep disorder. Symptoms of insomnia were reported by 78.2% of the population and 29.2% had moderate to severe insomnia. 78.4% of the population had poor sleep quality. Significant difference was noted among the different psychiatric groups when insomnia severity index (ISI. Population based studies have shown that psychiatric disorders and sleep disturbances are frequently reported together^2^^,^^3^. Of all psychiatric disorders, major depressive disorder, generalized anxiety disorder and alcohol use have been repeatedly found to be associated with insomnia^4^^-^^8^. Relationship between insomnia and depression has been found to be bidirectional and complex. Insomnia has been found to increase risk of incidental depression; has been reported as a residual symptom after remission of depression and lastly, both have been reported to wok in feed forward manner^6^^,^^9^. Contrarily, generalized anxiety disorder is known to increase chances of insomnia, while the opposite has not been reported^10^^,^^11^.

Similarly, use of addictive substances has been found to be associated with insomnia in multiple ways. Use of stimulating addictive substances e.g., nicotine may induce insomnia while withdrawal of substances with hypnotic potential e.g., alcohol is also known to cause insomnia^12^^-^^14^. It is not uncommon to have subjects who suffer from more than one of these conditions simultaneously. For example, use of addictive substances has been found to be associated with depression and generalized anxiety disorder^15^. Similarly, anxiety and depressive symptoms are co-morbid and share the neurobiological underpinnings^16^.

However, most of the data relating insomnia with psychiatric disorders have emerged from clinic-based studies. Epidemiological studies have been few and they have used different methodologies^2^^,^^3^^,^^17^^-^^20^. Data from the clinic based studies though partly represents the epidemiological patterns, still it can not be extrapolated to the population as the severity of symptoms is usually higher in subjects attending clinics. This becomes important especially in context of insomnia and depression as a dose related relationship may exist between them^21^.

Second, most of the studies have focused on relationship between insomnia and depression and. we could find only one study that has assessed relationship between anxiety and insomnia^2^^,^^18^^,^^19^^,^^22^^,^^23^. This study also focussed on trait anxiety rather than a specific anxiety disorder^23^. Third, as mentioned above, psychiatric disorders are often comorbid. However, in such cases which of the disorder has most pronounced effect on insomnia is not known. Considering these facts, present study was planned to assess prevalence of insomnia and commonly associated psychiatric disorders- depression, generalized anxiety disorder and addiction in Indian population. Another objective of the study was to find out relative effect of these disorders on insomnia.

## METHODS

This study was done after obtaining permission from institutional ethics committee. For this study, adult population residing in Doiwala block (rural) and wards of Rishikesh municipality of Dehradun district were chosen. This was a door to door survey using validated and translated questionnaires in Hindi. Sample size was calculated based on the prevalence of mental illnesses from a community-based study i.e. 6.1%^24^ and assuming 10% of non-response rate. The sample size came out to be 1693 and it was rounded off to 1700.

### Procedure

This cross-sectional population based study was conducted in the rural and urban areas of district Dehradun among individuals aged 20 years and above. Study spanned over a period of 12 months. The subjects were personally interviewed after obtaining written informed consent.

The desired sample size i.e. 1700 was distributed in rural & urban areas as per Probability Proportionate to Size (PPS) sampling, thus making the rural sample of 1098 and urban sample of 602^25^.

After line listing, the study houses were selected by systematic random sampling. In each selected household, all residents aged 20 years and above were listed and one individual was selected for the study by applying “Kish” method^26^. Sampled study subjects were informed about the purpose of the study and after obtaining their written informed consent, he/ she was interviewed.

The inclusion criteria for the study subjects were individuals aged 20 years and above, resident of the study area for a minimum of one year and ready to give consent, while those persons who did not fit in these criteria were excluded.

### Diagnosis of insomnia

Diagnosis of insomnia was made using Hindi version of Insomnia Severity Index (ISI)^27^^,^^28^. This is a seven item questionnaire that includes items related to initiation, maintenance of sleep and early morning awakening. Other items assess effect of insomnia on person’s daily activities and dissatisfaction with insomnia complaints. All items are scored on a five point Likert’s scale. Minimum score is 0 and maximum score is 28^27^. Hindi version has been found to be reliable with Cronbach’s alpha of 0.91^28^.

This scale has been found to be useful in diagnosing insomnia in primary care settings as well as in epidemiological studies^29^^,^^30^. However, different cut-off scores have been reported to detect clinical insomnia- 10 in the epidemiological study and 14 for the patients attending primary care facility^29^^,^^30^. Since, normative data was not available for Indian population, we followed the scoring system as provided by the authors of original study- score between 0-7 depicts no insomnia, between 8-14 depicts subthreshold insomnia, between 15-21 moderate insomnia and score above 21 depicts severe insomnia. For present study, score above 7 was considered as clinical insomnia.

### Diagnosis of Psychiatric Disorders

Hindi version of MINI 6.0.0 was used after obtaining permission from one of the authors^31^. This structured Psychiatric interview is based upon ICD-10 criteria. For the present study, sections of mood disorders, generalized anxiety disorder and substance use were used. All interviews were done by one of the authors (IW). This structured interview was used to diagnose major depressive disorder- current episode, past episode and recurrent episodes (module A); hypomania and mania: present and past episodes (module C); alcohol abuse and dependence: present episode (module I); substance use and substance dependence: current episode that includes cannabis, stimulants, benzodiazepines to name a few (module J) and generalized anxiety disorder current episode (module N).

### Diagnosis of tobacco use

Tobacco use was ascertained by obtaining history from the subjects. They were asked about the form of tobacco that they were using- chewable, smoke or sniffing. In addition, duration and frequency of the use was also asked. However, information regarding withdrawal symptoms and dependence could not be reliably obtained. Hence, diagnosis of “tobacco use” was made.

### Statistical Analysis

Statistical analysis was done using SPSS v. 21.0 (IBM SPSS Statistics for Windows, Version 21.0. Armonk, NY: IBM Corp.). Chi square analysis was used to compare significance of proportions. Statistical significance of numerical data between two groups was analyzed using independent sample t test. Binary logistic regression was run using the variables that were found significant in univariate analyses comparing subjects with and without insomnia. Three models were run, first with lifetime episode of major depressive disorder, second that included current episode of major depressive episode along with other variables and third, including socio-demographic variables as depicted in Table 1.

**Table 1:** Comparison of subjects with and without insomnia*.

S.N.	Character	Insomnia			*p*
		Present (n=175)	Absent (n=1525)	Total	
1.	Gender				
	Male	98 (56%)	773 (50.7%)	871 (51.2%)	
	Female	77 (44%)	752 (49.3%)	829 (48.8%)	0.18
2.	Age (years)	49.16 + 14.23	38.23 + 13.45		<0.001
3.	Residence				
	Urban	75 (42.9%)	226 (14.8%)	301 (17.7%)	
	Peri-urban	35 (20%)	266 (17.4%)	301 (17.7%)	
	Rural	65 (37.1%)	1033 (67.7%)	1098 (64.6%)	<0.001
4.	Education				
	None	33 (18.9%)	449 (29.4%)	482 (28.4%)	
	High School (8 years)	30 (17.1%)	296 (19.4%)	326 (19.2%)	
	Intermediate (12 years)	69 (39.4%)	479 (31.4%)	548 (32.2%)	
	Graduate and above	43 (24.6%)	301 (19.7%)	344 (20.2%)	0.009
5.	Occupation				
	Not working	86 (49.1%)	828 (54.3%)	914 (53.8%)	
	Serivce	56 (32%)	406 (26.6%)	462 (27.2%)	
	Self employed	33 (18.8%)	291 (19.1%)	324 (19%)	0.06
6.	Marital Status				
	Unmarried	9 (5.1%)	242 (15.9%)	251 (14.8%)	
	Married	141 (80.6%)	1228 (80.5%)	1369 (80.5%)	
	Separated	25 (14.3%)	55 (3.6%)	80 (4.7%)	<0.001
7.	Family Type				
	Joint	85 (48.6%)	471 (30.9%)	556 (32.7%)	
	Nuclear	90 (51.4%)	1054 (69.1%)	1144 (67.3%)	<0.001
8.	Over crowding				
	Present	108 (61.7%)	883 (57.9%)	991 (58.3%)	
	Absent	67 (38.3%)	642 (42.1%)	709 (41.7%)	0.33
9.	Socio-Economic Class				
	Upper	4 (2.3%)	47 (3.1%)	51 (3%)	
	Middle	118 (67.4%)	745 (48.9%)	863 (50.8%)	
	Lower	53 (30.3%)	733 (48.1%)	786 (46.2%)	<0.001
10.	Major Depressive Episode				
	Present	21 (12%)	70 (4.6%)	91 (5.4%)	
	Absent	154 (88%)	1455 (95.4%)	1609 (94.6%)	<0.001
11.	Generalized Anxiety				
	Disorder	49 (28%)	21 (1.4%)	70 (4.1%)	
	Present	126 (72%)	1504 (98.6%)	1630 (95.9%)	<0.001
	Absent				
12.	Alcohol Dependence				
	Present	40 (22.9%)	173 (11.3%)	213 (12.5%)	
	Absent	135 (77.1%)	1352 (88.7%)	1487 (87.5%)	<0.001
13.	Cannabis Dependence				
	Present	10 (5.7%)	41 (2.4%)	47 (2.8%)	
	Absent	165 (94.3%)	1484 (97.6%)	1653 (97.2%)	0.02
14.	Tobacco use	77 (44%)	289 (19%)	366 (21.5%)	
	PresentAbsent	98 (56%)	1236 (81%)	1334 (78.5%)	<0.001

## RESULTS

Genders represented almost equally in this study (male 51.2%), 71.4% were literate and 80.5% were married. Nearly two third were living in nuclear families and 58.3% reported overcrowding in the house.

Psychiatric disorders were reported by 20.5% population. Life-time prevalence of major depressive episode was 5.4% while point prevalence was 3.6%. Lifetime prevalence of manic episode was 0.1%. Point prevalence of generalized anxiety disorder was 4.1%. Interestingly, tobacco was the most commonly used addictive substance (21.5%) followed by alcohol. 12.5% met the criteria for alcohol dependence while alcohol abuse was reported by 3.5% subjects. 3% reported cannabis dependence, while 1.1% had cannabis abuse.

Binary logistic regression analysis was done with the variables that were found significant in univariate analysis. First model included age, lifetime episode of major depressive disorder, generalized anxiety disorder, tobacco use, cannabis dependence and alcohol dependence. It classified 91.4% cases correctly and this model was overall significant (*p*<0.001). This model explained for 29% variance of the included variables (Negelkerke R square 0.286) (Table 2). Second model that included current episode of major depressive disorder classified 91.3% cases correctly and was overall significant (*p*<0.001). It explained 28% variance of the factors (Negelkerke R square 0.285) (Table 2). In model 3, all sociodemographic variables were also included. This classified 91.6% cases correctly and explained 37% variance. It was overall significant (*p*<0.001).

**Table 2:** Binary Logistic Regression Analysis depicting factors associated with Insomnia.

S.N.	Variable	B	SE	*p*	OR	95% CI	
						Lower	Upper
*Model 1*:							
1.	Age (years)	0.047	0.006	<0.001	1.04	1.03	1.06
2.	Alcohol Dependence	0.30	0.23	0.19	1.35	0.85	2.15
3.	Cannabis Dependence	-1.04	0.50	0.03	0.35	0.13	0.94
4.	Tobacco use	0.93	0.20	<0.001	2.55	1.72	3.77
5.	Generalized Anxiety	3.41	0.33	<0.001	30.46	15.73	58.99
6.	Major Depressive Disorder Life time episodes	-0.49	0.37	0.18	0.60	0.29	1.26
*Model 2:*							
1.	Age (years)	0.046	0.006	<0.001	1.04	1.03	1.06
2.	Alcohol Dependence	0.32	0.23	0.18	1.37	0.86	2.16
3.	Cannabis Dependence	-1.07	0.51	0.03	0.34	0.13	0.92
4.	Tobacco use	0.93	0.20	<0.001	2.53	1.71	3.74
5.	Generalized Anxiety	3.35	0.32	<0.001	28.58	15.15	53.90
6.	Major Depressive Disorder Current episode	-0.49	0.42	0.25	0.61	0.26	1.41
*Model 3:*							
	Age (in years)	0.05	0.008	<0.001	1.05	1.04	1.07
	Education^1^			<0.001			
	No Education	-2.30	0.36	<0.001	0.10	0.04	0.20
	High School	-0.94	0.31	0.003	0.38	0.20	0.72
	Intermediate	-0.23	0.24	0.35	0.79	0.48	1.29
	Occupation^2^						
	Service	-0.68	0.26	0.008	0.50	0.30	0.83
	Agriculture	0.04	0.37	0.90	1.04	0.50	2.17
	Self Employed	-0.78	0.35	0.03	0.45	0.22	0.92
	Socio-economic Status^3^						
	Upper	-0.38	0.62	0.54	0.68	0.19	2.33
	Middle	0.361	0.22	0.10	1.43	0.92	2.21
	Marital Status^4^						
	Married	-0.55	0.38	0.15	0.57	0.27	1.22
	Unmarried	-1.46	0.60	0.01	0.23	0.07	0.76
	Over Crowding Present	0.24	0.21	0.25	1.27	0.84	1.93
	Major Depressive Episodes Lifetime	-0.31	0.41	0.43	0.72	0.32	1.62
	Generalized anxiety disorder	3.22	0.35	<0.001	25.26	12.54	50.85
	Alcohol Dependence	-0.045	0.27	0.86	0.95	0.56	1.62
	Tobacco Use	1.44	0.24	<0.001	4.22	2.63	6.78
	Cannabis Dependence	-0.96	0.53	0.07	0.38	0.13	1.09

Reference categories 1.Graduate and above; 2 Unemployed; 4 Separated; 4 Lower

## DISCUSSION

In this study clinical insomnia was reported by one tenth of the adult population. This study also showed that in increasing age, higher education, umemployment, tobacco use and generalized anxiety disorder increased the odds for having clinical insomnia. Among these, older age is an established risk factor for clinical insomnia^32^. However, other four findings (tobacco use, generalized anxiety disorder, unemployment and higher education) are novel as they have not been investigated general population, to best of our knowledge. Even more interesting was the fact that in multivariate analysis, socio-demographic factors like socio-economic status, marital status, family type, crowding, major depressive disorder, and alcohol dependence were excluded from the model, many of which are established risk factor for insomnia.

In previous epidemiological studies, prevalence if insomnia has been reported to vary between 10.2-19.7%^2^^,^^9^^,^^23^^,^^33^. This difference is related to difference in methodology of the study. For example, studies using sleep diary^23^ reported higher prevalence compared to those using structured interview^2^. Other factors e.g., criteria followed to diagnose insomnia, characteristics of population included viz., age, race, gender, use of addictive substances and comorbidities also influenced the results of previous studies^32^. In present study, ISI was used to diagnose insomnia, which contained items related to initial, middle and terminal insomnia as well as effect of insomnia on daytime functions^27^^,^^28^. It assessed symptoms within a span of past seven days, which was contrary to the present definition of insomnia that focuses on frequency as well as duration of symptoms^32^.

A recent meta-analysis reported that ISI could be used as a reliable tool to diagnose insomnia with 88% sensitivity and 81% specificity, substantiating the results of present study^34^. Another issue is- whether objective methods would have influenced the results of the study? In one of the earlier studies, objective assessment had shown higher prevalence of insomnia, compared to subjective report, which is contrary to the standard definition of insomnia, which focuses on subjective report^32^^,^^33^. Hence, we opine that results of present study are reliable.

Contrary to the established fact that females are predisposed to insomnia, we did not find effect of gender in present study on prevalence of insomnia^32^. In earlier studies, female preponderance for insomnia has been ascribed to multiple factors e.g., genetic factors, predisposition towards anxiety, depression and coping styles to name a few^23^. However, this data was from western population and a meta-analysis has also reported male predisposition for insomnia in East Asian population^35^. Though the reasons are not entirely clear, it is possible that other factors interacted with gender to nullify the gender predisposition in present study. For example, three factors- increasing years of education, urban population and middle socio-economic group had higher prevalence of insomnia in this study.

It is possible that these factors exposed male subjects to emotional stresses, most of who were bread-earners for their family compared to female subjects who were largely home-makers. Gender difference in coping-skills is known with women more frequently using socialization while men focus on the problem and may get indulged in rumination^36^. Rumination related to daytime issues has been reported in this population when they were not able to fall asleep, and can perpetuate insomnia by increasing stress, as described in stress-diathesis theory of insomnia^32^^,^^37^. Other possibility could be related to use of addictive substances like tobacco and alcohol, which are culturally more acceptable among males. This could have increased the rates of insomnia among males leading to disappearance of gender effect. Lastly, in the Indian society, males are mainly responsible for providing financial support to the family, and high rates of unemployment, as discussed above could have led to higher prevalence of insomnia among males.

Higher education and unemployment were associated with insomnia in present study. However, previous studies have shown higher prevalence of insomnia among subjects with lower education and among unemployed subjects^38^^,^^39^. Difference in the results of the present study could be related to high proportion of unemployment seen among subjects with graduation or higher education. This could have led to stress and consequent insomnia. Consistent with the results of present study, role of socio-economic variables is unclear with one study showing increased prevalence of insomnia among socially disadvantaged persons, while other showing inconsistent relationship^38^^,^^39^. Interestingly, type of family and crowding were not associated with insomnia in this study.

Previous data have shown that single women parents have higher risk of having insomnia, whereas two-parents living in a nuclear family have lesser chances to have insomnia^40^^,^^41^. Joint families may improve the sleep by sharing the household responsibilities among members, however, this effect may be culture specific. In traditional Indian setting, younger women in the joint family carry the burden of household work, that of child-care as well as care of elders. These responsibilities increase the stress and could be one reason why we did not find any difference between family types. Though theoretically, crowding in the bedroom may impair sleep quality, yet we did not find any effect of overcrowding in this study. In Indian settings, bed-room is usually shared among parents and young children. Most of people grow with this habit and hence, it may not impair their sleep. This is in concordance with results of previous study^42^. However, these findings need to be examined in future.

Insomnia has been found to be associated with clinically significant depression as well as anxiety in previous studies^19^^,^^23^. However, it must be remembered that methodology was different across studies. For diagnosis of depression different methods have been used e.g., Beck’s depression inventory (BDI)^23^ or patient health questionnaire-9 (PHQ-9)^19^. Among these, BDI contains items related to somatic as well as cognitive components, both of which are seen in subjects with insomnia. This can lead to spurious diagnosis of depression in these patients^32^. On the other hand, MINI focuses on cardinal (i.e., emotional) symptoms of depression. This could have led to exclusion of cases with prominent somatic and cognitive symptoms that are usually seen as daytime manifestations of insomnia. This could be one reason, why we did not find association between major depressive disorder and insomnia in present study in all models (Table 2).

This finding is reiterated by studies which reported improvement in depressive symptoms after adequate management of insomnia^43^. Secondly, most of the studies have focused on depression and did not assess anxiety concurrently, thus comparative effect of anxiety and depression could not be assessed^2^^,^^19^. van Mill et al.^44^ assessed both major depressive disorders as well as generalized anxiety disorder and reported that both disorders increased odds for insomnia. Among all anxiety disorders, only subjects with generalized anxiety disorder have shown the polysomnographic evidence of disturbed sleep^45^^,^^46^. Still, there is dearth of literature that has assessed effect of comorbid depression and anxiety disorders on insomnia, and this study was an attempt to fill that gap^46^.

Alcohol dependence did not influence insomnia in this study while tobacco use increased the odds for insomnia. Though the insomnia was more prevalent in subjects with alcohol dependence in univariate analysis, as seen in a previous studies^47^^,^^48^, this effect disappeared during multivariate analysis. In Indian clinical settings, it has been observed that most of the patients use nicotine in any form (chewing or smoking) after taking alcohol. It is possible that insomnia in these subjects is actually related to nicotine rather than alcohol, as seen in present as well as earlier study^48^. In addition, psychiatric disorders have been found to influence occurrence of insomnia among subjects with alcohol dependence in various studies, similar to the findings of the present study^48^^,^^49^.

It must be noted that previous studies assessing association between insomnia and alcohol had different methodology compared to present study. Their cohort was subjects having alcohol related disorders, on the contrary, present study was population based; hence, head to head comparison of these studies was not possible. Another interesting finding was lesser prevalence of insomnia among cannabis users. This could be related to pharmacological properties of cannabis and requires further investigation. Nabinol and cannabinol, both of which are cannabinoids are known to reduce sleep onset latency and relieve obstructive sleep apnea; contrarily, cannabis withdrawal is associated with sleep disruption^50^^,^^51^. However, effect of cannabis disappeared when socio-economic variables were entered (model 3, Table 2) suggesting an interaction between cannabis and socio-demographic factors, which requires further investigation.

Like any other scientific investigation, this study also had some methodological limitations. First, diagnosis of insomnia as well as psychiatric disorders was made using structured interview rather than clinical interview. However, measures that were used have optimal psychometric properties making them suitable for epidemiological study, as mentioned in methodology. Second, because of the study design we could not assess differential effect of amount and frequency of addictive substances on prevalence of insomnia. This is an area worth investigating and will be assessed in future. Third, we did not rule out effect of other disorders that may interfere with sleep e.g., circadian rhythm sleep disorder, sleep apnea and restless legs syndrome. Fourth, other medical disorders e.g., congestive heart failure and chronic pain to name a few, are known to be associated with sleep disturbances. They were not addressed in the present study.

In conclusion, this study showed that insomnia affects around 10% of the population. It was associated with unemployment, higher education, generalized anxiety disorder and tobacco use in the community based sample.
